# Neoadjuvant PD-1/PD-L1 combined with CTLA-4 inhibitors for solid malignancies: a systematic review and meta-analysis

**DOI:** 10.1186/s12957-023-03212-5

**Published:** 2023-11-06

**Authors:** Shuang Huang, Gang Zheng, Kai Yang

**Affiliations:** 1 Department of Stomatology, Shapingba Hospital affiliated to Chongqing University, No.44, Xiaolongkan New Street, Chongqing, Shapingba District 400030 China; 2 Anorectal Department, Chongqing Traditional Chinese Medicine Hospital, 6 Panxi 7 Road, Jiangbei District, Chongqing, 400021 China; 3 Department of Oral and Maxillofacial Surgery, The First Affiliated Hospital of Chongqing Medical University, No.1, Youyi Road, Yuzhong District, Chongqing, 400016 China

**Keywords:** Neoadjuvant therapy, Immune checkpoint inhibitors, PD-1, PD-L1, CTLA-4, Meta-analysis

## Abstract

**Background:**

The effectiveness and safety of neoadjuvant PD-1/PD-L1 inhibitors combined with CTLA-4 inhibitors is controversial. This systematic review and meta-analysis aimed to evaluate the efficacy and safety of PD-1/PD-L1 inhibitors combined with CTLA-4 inhibitors as neoadjuvant therapy for malignant solid tumors.

**Methods:**

This study has been registered with the number CRD42023407275 on PROSPERO. Systematic searches were conducted in PubMed, Embase, Web of Science and Cochrane Library databases until March 17, 2023. In addition, manual searches were performed. The inclusion criteria encompassed randomized controlled trials (RCTs) that assessed the utilization of neoadjuvant PD-1/PD-L1 inhibitors combined with CTLA-4 inhibitors PD-1/PD-L1 inhibitors for patients with solid malignancies. The Cochrane Collaboration's tool for assessing risk of bias in randomized trials (ROB1) were used. Risk ratios (RRs), hazared ratios (HRs) and their respective 95% confidence intervals were calculated using Stata17.0 MP and Review Manager 5.4 software.

**Results:**

A total of 2780 records were identified, and ultimately 10 studies involving 273 patients were included. The meta-analysis showed that the addition of CTLA-4 inhibitors to PD-1/PD-L1 inhibitors did not demonstrate a significant effect on overall response rate, main pathological response, pathological complete response, surgical resection, radical resection, overall survival, progression-free survival, recurrence-free survival, grade 3–4 adverse events, all-cause mortality, and completed treatment (*P* > 0.05). However, further subgroup analysis indicated that the combination of PD-1 with CTLA-4 inhibitors significantly increased the occurrence of grade 3–4 adverse events in patients (*P* < 0.05).

**Conclusions:**

As neoadjuvant therapy for malignant solid tumors, the addition of CTLA-4 inhibitors to PD-1/PD-L1 inhibitors does not appear to enhance efficacy.Moreover, there is a potential increase in the risk of grade 3–4 adverse events associated with this combination. However, it is important to note that the studies included in this analysis suffer from limitations such as small samples and single-center designs, which are inherent constrains with the available published literature. Further research involving large-sample and multicenter RCTs are warranted to obtain more reliable results.

**Supplementary Information:**

The online version contains supplementary material available at 10.1186/s12957-023-03212-5.

## Introduction

In 70 countries, cancer is the second leading cause of death following cardiovascular diseases. Remarkably, in 57 countries cancer has even surpassed cardiovascular disease to become the top spot in mortality among humans [1]. Nearly 10 million people worldwide died of cancer in 2020 [2]. Therefore, conquering cancer assumes paramount importance as it directly contributes to prolonging human lifespan and enhancing the quality of life. The discovery and clinical application of PD-1 (Programmed cell death 1), PD-L1 (Programmed cell death ligand 1), and CTLA-4 (Cytotoxic T lymphocyte antigen 4) and their inhibitors have introduced novel therapeutic approaches for combating malignant tumors [3–7].

PD-1/PD-L1 and CTLA-4 inhibitors fuction through distinct pathways. The PD-1/PD-L1 inhibitor operates by blocking the interaction between PD-1 located on T cell membranes and the overexpressed PD-L1 on cancer cell membranes, while CTLA-4 inhibitor works by blocking the binding of B7 on antigen-presenting cells to CTLA-4 in T cells [8, 9]. Monotherapy with PD-1 or PD-L1 inhibitors is limited for patients with solid malignancies, and new strategies are required [10]. Combining the CTLA-4/B7 axis blockade, as an auxiliary axis, with PD-1/PD-L1 axis blockade, has become a new direction of cancer immunotherapy [11, 12]. Several studies have demonstrated that the addition of a CTLA-4 inhibitor to PD-1/PD-L1 inhibitors benefits for patients with recurrent/metastatic solid malignancies [13–18]. In addition, results from randomized controlled trials (RCTs) have been published that compare the use of neoadjuvant PD-1/PD-L1 inhibitors alone with the combination of neoadjuvant PD-1/PD-L1 inhibitors and CTLA-4 inhibitors. However, these RCTs suffer from small sample sizes, scattered sites, and inconsistent results [19–21]. Furthermore, no related secondary studies have been published. Therefore, a systematic review and meta-analysis is needed to evaluate the effectiveness and safety of neoadjuvant therapy with CTLA-4 inhibitors added to PD-1/PD-L1 blockade for solid malignancies.

In accordance with the PICOS principle, we used overall response rate (ORR), main pathological response (MPR), pathological complete response (pCR), surgical resection, radical resection (R0 resection), overall survival (OS), progression-free survival (PFS) and recurrence-free survival (RFS) as the efficacy outcomes (O), and grade 3–4 adverse events (grade 3–4 AEs), all-cause mortality and completed treatment as the safety outcomes (O). We conducted a meta-analysis focusing on published RCTs (study design, S) comparing the efficacy and safety of the neoadjuvant combination therapy of PD-1/PD-L1 inhibitors and CTLA-4 inhibitors (intervention, I) with neoadjuvant monotherapy of PD-1/PD-L1 inhibitors (comparison, C) for patients with solid malignancies (population, P) to provide theories for clinical applications or futural investigations.

## Methods

### Literature search strategy

This study was conducted in accordance with the Preferred Reporting Items for Systematic Reviews and Meta-Analyses (PRISMA) guidelines [22] and has been registered with the number CRD42023407275 on PROSPERO. Electronic databases such as PubMed, Embase, Web of Science, and the Cochrane Library were searched for records published from inception to March 17, 2023. Searches were conducted using using the following keywords with their subject terms and free words: “PD-1”, “PD-L1”, “CTLA-4”, “immunotherapy”, “neoadjuvant”, “cancer”, and “randomized controlled trial”. And the ClinicalTrial.gov registered website, European Society of Clinical Oncology, American Society of Clinical Oncology conference abstracts within the past 5 years, and references of all included articles were also manually searched (Table S1). Two authors (SH and GZ) performed the search independently and disagreements were resolved by consultation.

### Inclusion and exclusion criteria

The inclusion criteria were as follows: (1) patients with solid malignancies diagnosed by histopathology; (2) without distant metastasis; (3) RCTs; (4) neoadjuvant PD-1 or/and PD-L1 inhibitor combined with CTLA-4 inhibitor were used for experimental groups; (5) neoadjuvant PD-1 or/and PD-L1 inhibitor was used for control groups; (6) at least one of the following outcomes was available: ORR, MPR, pCR, surgical resection, R0 resection, OS, PFS, RFS, grade 3–4 AEs, all-cause mortality and completed treatment; and (7) English publications.

The exclusion criteria were as follows: (1) reviews, systematic reviews, meta-analyses, letters, case reports, and public database analyses; (2) vitro and experimental animal studies; (3) unavailable outcomes; (4) less than five cases; and (5) duplicate studies. The most complete and latest articles were included, if duplicate reported cases were involved in different articles.

### Data extraction

The process of data extraction was conducted independently by two authors (SH and GZ) according to the guide tables. The following information was extracted: authors, years, registration numbers, cancers, drugs administered in experimental and control groups, number of participants, and outcomes. The number of events and non-occurred events in experimental and control groups were extracted for ORR, MPR, pCR, surgical resection, R0 resection, grade 3–4 AEs, all-cause mortality, and completed treatment. Hazard ratios (HRs) and their 95% confidence interval (CI) were extracted for OS, PFS, and RFS. If HRs were not reported, we used Engauge Digitizer 4.1 software [23] and the method introduced by Jayne F Tierney [24] to extract the HR and 95% CI.

### Data analysis

This meta-analysis was conducted using Stata17.0 MP and Review Manager 5.4. Heterogeneity among the included studies was assessed with the χ^2^ test and I^2^ test. Studies were considered heterogenous if *P* ≥ 0.1 and I^2^ ≤ 50%, and meta-analysis were performed using a fixed-effects model. On the contrary, a random-effect model was used when heterogeneity was observed. The Cochrane Risk of Bias Assessment Tool (ROB1) was used to assess the risk of biases in the included articles. Risk ratios (RRs) and their 95% CIs were calculated for ORR, MPR, pCR, surgical resection, R0 resection, grade 3–4 AEs, all-cause mortality, and completed treatment. HRs and their 95% CIs were calculated for OS, PFS, and RFS. Stability was assessed by sensitivity analysis, and further subgroup analyses were performed based on the use of PD-1 or PD-L1 inhibitors in control groups. Egger and begg test were used to evaluate publication biases. The test level was *P* = 0.05.

## Results

### Features and systematic review of the included studies

**Fig. 1:**
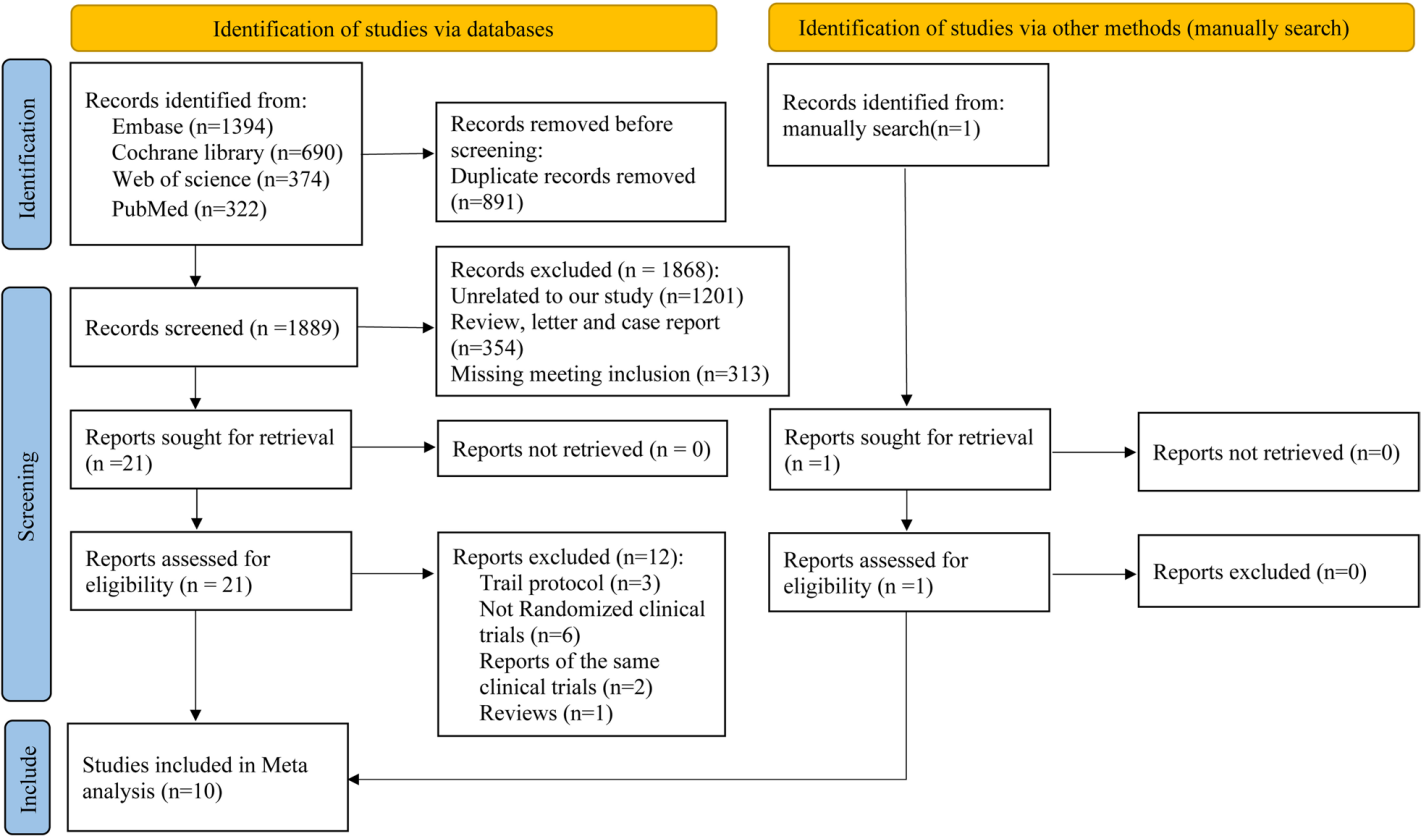
Flowchart of the literature research process

Detailed steps during the literature research are described in Fig. 1. A total of 2780 potential records were identified, and 891 duplicate records were removed. Finally, 10 studies [19–21, 25–31] were included in our study that met the criteria. Included articles were single-center RCTs published from November 2018 to January 2023. These articles consisted of seven full-text articles, two conference abstracts, and one clinical trial result report. Six studies used a combination of PD-1 inhibitors and CTLA-4 inhibitors, while four studies utilized PD-L1 inhibitors in combination with CTLA-4 inhibitors. Notably, none of the included studies used both PD-1 and PD-L1 inhibitors simultaneously. Four articles focused on head and neck squamous cell carcinoma, two on non-small cell lung cancer, one on pancreatic cancer, one on ovarian cancer, one on melanoma, and one on malignant pleural mesothelioma. A total of 273 participants were included in the study, with 137 participants in the experimental groups and 136 participants in the control groups. Table 1 summarized the specific features of the included articles. The assessment of bias is shown in Fig. S1.

**Table 1:** Features of the included studies

No	Author	Year	NCT	cancers	durgs	Pts	follow-up (months)	Effecacy	Safety
1	Rodabe N. Amaria	2018	NCT02519322	Melanoma	Anti-PD-1 VS. Anti-PD-1 + Anti-CTLA-4	23	15.0 vs 15.6	ORR, pCR, surgery rate, OS, PFS, RFS	3–4 grade AEs
2	Boris Sepesi^a^	2022	NCT03158129	NSCLC	Anti-PD-1 VS. Anti-PD-1 + Anti-CTLA-4	44	NA	surgery rate, R0 rate	all-cause mortality
3	Tina Cascone^a^	2021	NCT03158129	NSCLC	Anti-PD-1 VS. Anti-PD-1 + Anti-CTLA-4	44	average 22.2	ORR, MPR, pCR, OS, RFS	3–4 grade AEs, completed therapy
4	Jonathan D Schoenfeld^b^	2020	NCT02919683	OSCC	Anti-PD-1 VS. Anti-PD-1 + Anti-CTLA-4	29	average 14.2	ORR, MPR, pCR, OS, PFS	3–4 grade AEs, completed therapy
5	Jonathan D Schoenfeld^b^	2022	NCT02919683	OSCC	Anti-PD-1 VS. Anti-PD-1 + Anti-CTLA-4	29	NA	NA	updated completed therapy, all-cause mortality
6	Renata Ferrarotto	2020	NCT03144778	OPSCC	Anti-PD-L1 VS. Anti-PD-L1 + Anti-CTLA-4	28	average 15.79	ORR, MPR, pCR	3–4 grade AEs
7	Ahmed Omar Kaseb	2022	NCT03222076	HC	Anti-PD-1 VS. Anti-PD-1 + Anti-CTLA-4	27	NA	ORR, pCR, PFS, surgery rate	3–4 grade AEs, all-cause mortality
8	A. Leary	2021	NCT03249142	OC	Anti-PD-L1 VS. Anti-PD-L1 + Anti-CTLA-4	66	NA	MPR, surgery rate, R0 rate	3–4 grade AEs, completed therapy
9	Hye Ryun Kim	2021	NCT03737968	HNSCC	Anti-PD-L1 VS. Anti-PD-L1 + Anti-CTLA-4	36	average 4.3	ORR	NA
10	Hyun-Sung Lee	2023	NCT02592551	MPM	Anti-PD-L1 VS. Anti-PD-L1 + Anti-CTLA-4	20	average 34.1	MPR、AEs	NA

*Abbreviation*: *No.* Number, *Pts* Participants, *NSCLC* Non-small cell cancer, *OSCC* Oral squamous cell carcinoma, *OPSCC* Oropharyngeal squamous cell carcinoma, *HC* Hepatic cancer, *OC* Ovarian cancer, *HNSCC* Head and neck squamous cell carcinoma, *MPM* Malignant pleural mesothelioma, *NA* Not applicable, *ORR* Overall response rate, *MPR* Main pathological response, *pCR* Pathological complete response, *R0* Radical resection, *OS* Overall survival, *PFS* Progression-free survival, *RFS* Recurrence-free survival, *AEs* Adverse event rate

^a^Assessed the same clinical trial (NCT03158129) with different outcomes

^b^Assessed the same clinical trial (NCT02919683) with different outcomes

### Meta-analysis

#### ORR, pCR and MPR

**Fig. 2:**
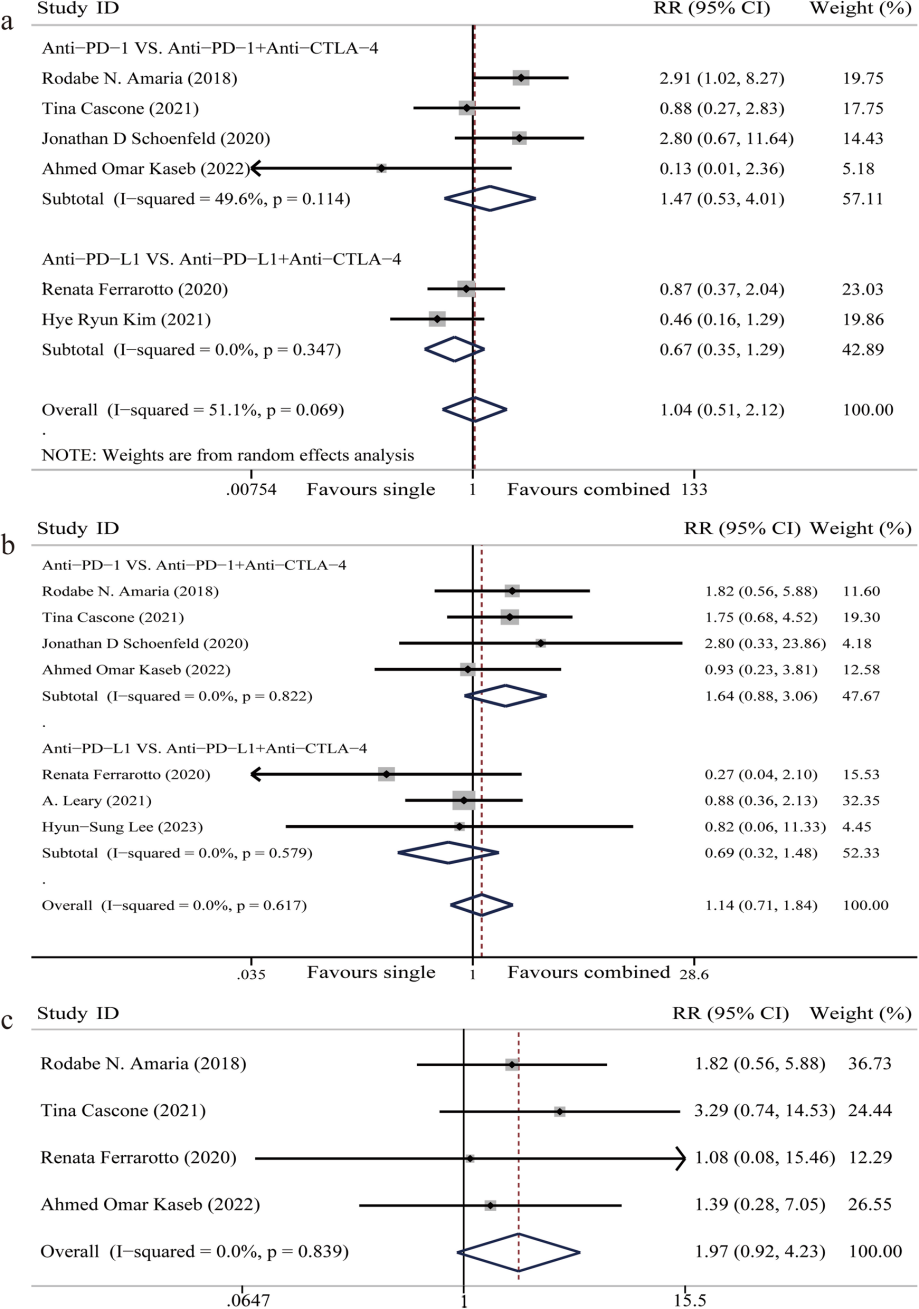
Forest plot of overall response rate (ORR), main pathological response (MPR), and pathological complete response (pCR). **a** Forest plot of ORR. **b** Forest plot of MPR. **c** Forest plot of pCR

ORR: Six articles reported on ORR, involving 187 participants [19–21, 26, 28, 30]. The random-effect model was used because of significant heterogeneity among studies (I^2^ = 51.1%). Meta-analysis showed that there was no significant difference in ORR when CTLA-4 inhibitors were added to neoadjuvant PD-1/PD-L1 inhibitor for patients with solid malignancies (RR 1.04, 95%CI 0.51–2.12, *P* = 0.91) (Fig. 2a). Subgroup analysis indicated that the addition of CTLA-4 inhibitors had no significant impact on ORR regardless of whether PD-1 or PD-L1 inhibitor were used (*P* > 0.05) (Fig. 2a).

MPR: Seven articles reported on MPR, involving 232 participants [19–21, 26, 28, 29, 31]. The fixed-effect model was used because of homogeneity among studies (I^2^ = 0%). Meta-analysis showed that there is no significant difference in MPR when CTLA-4 inhibitors were added to neoadjuvant PD-1/PD-L1 inhibitor for patients with solid malignancies (RR 1.14, 95%CI 0.71–1.84, *P* = 0.58) (Fig. 2b). Subgroup analysis showed that the addition of CTLA-4 inhibitors had no significant impact on MPR regardless of whether PD-1 or PD-L1 inhibitor were used (*P* > 0.05) (Fig. 2b).

pCR: Four articles reported on pCR, involving 119 participants [20, 21, 26, 28]. The fixed-effect model was used because of homogeneity among studies (I^2^ = 0%). Meta-analysis showed that the additional CTLA-4 inhibitors had no significant impact on pCR (RR 1.97, 95%CI 0.92–4.23, *P* = 0.08) (Fig. 2c).

#### Surgical resection

**Fig. 3:**
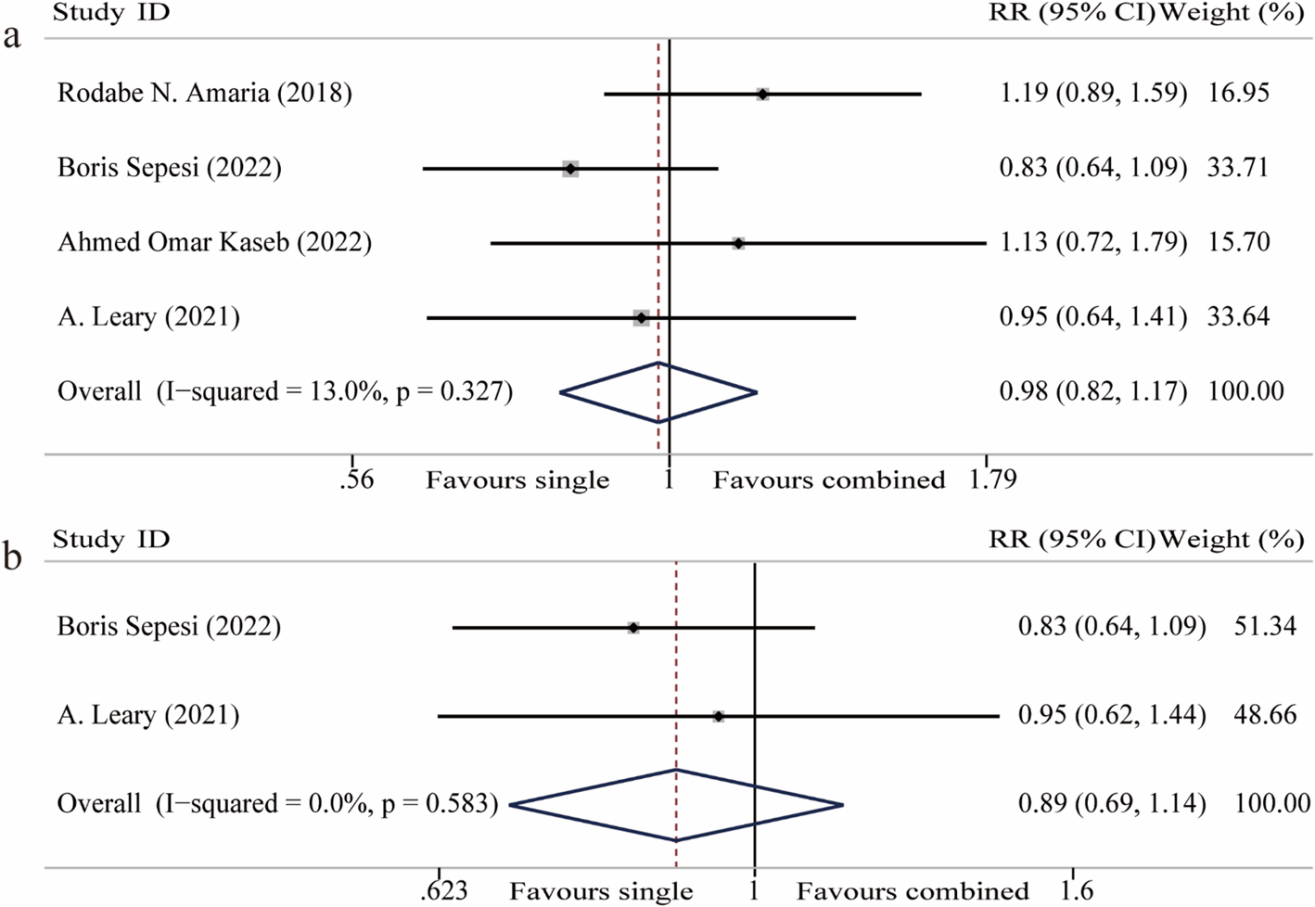
Forest plot of surgical resection and residual 0 resection (R0). **a** Forest plot of surgical resection. **b** Forest plot of R0 resection

Four articles reported on surgical resection, involving 158 participants [20, 21, 25, 29]. The fixed-effect model was used because of homogeneity among studies (I^2^ = 13.0%). Meta-analysis showed that there is no significant difference in surgery when CTLA-4 inhibitors were added to neoadjuvant PD-1/PD-L1 inhibitor for patients with solid malignancies (RR 0.98, 95%CI 0.82–1.17, *P* = 0.83) (Fig. 3a).

Two of those four articles further reported on R0 resection. The fixed-effect model was used because of homogeneity among studies (I^2^ = 0%). Meta-analysis showed that the addition of CTLA-4 inhibitors had no significant impact on R0 resection (RR 0.89, 95%CI 0.69–1.14, *P* = 0.36) (Fig. 3b).

#### Survivals

**Fig. 4:**
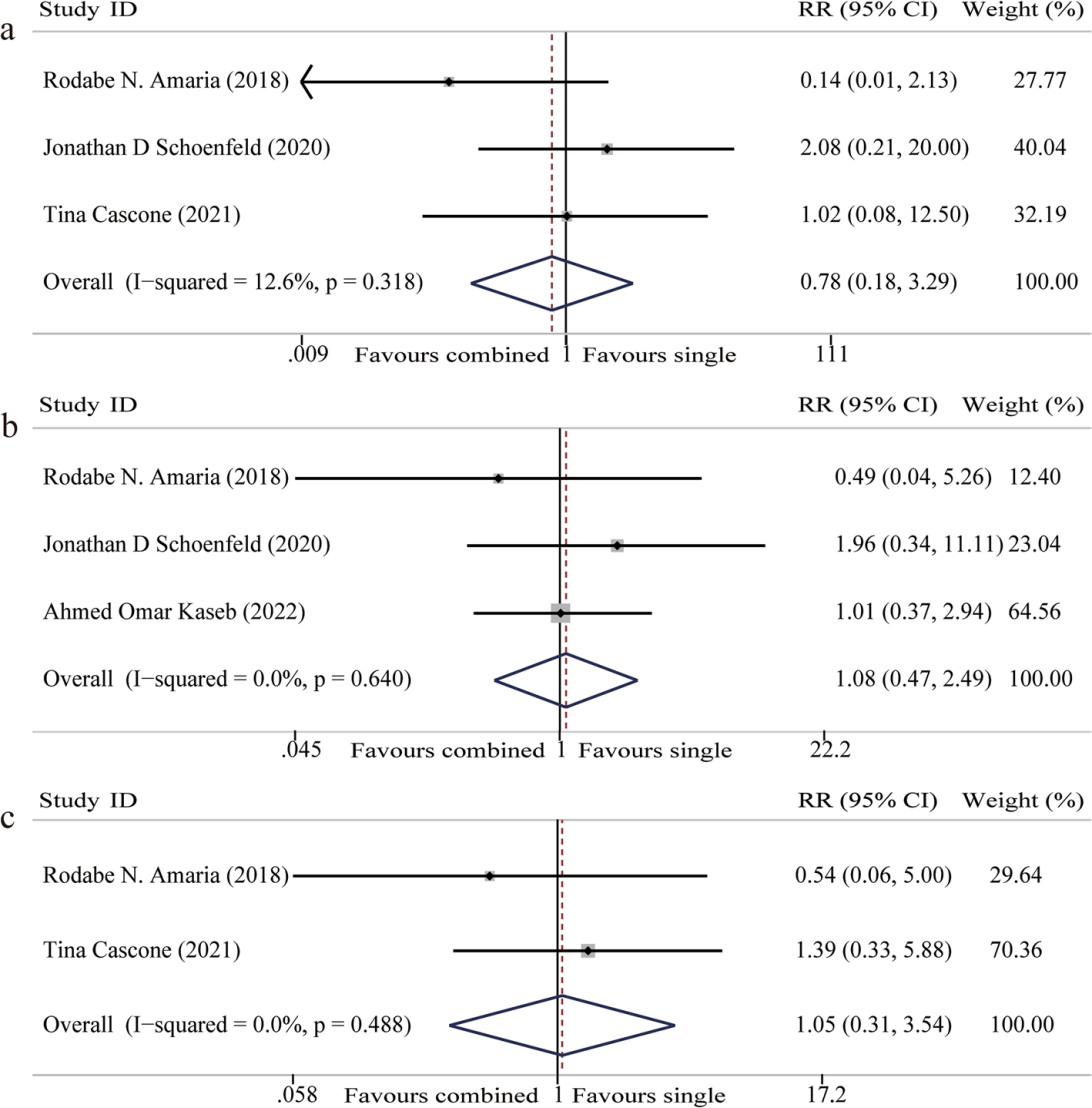
Forest plot of overall survival (OS), progression-free survival (PFS), and recurrence-free survival (RFS). **a** Forest plot of OS. **b** Forest plot of PFS. **c** Forest plot of RFS

OS: OS was reported in three studies, involving 96 participants [19, 21, 26]. The fixed-effect model was used because of homogeneity among studies (I^2^ = 12.6%). Meta-analysis showed that the addition of CTLA-4 inhibitors had no significant impact on prolonging OS of patients (HR 0.78, 95% CI 0.18–3.29, *P* = 0.74) (Fig. 4a).

PFS: Three studies reported on PFS, involving 79 participants [19–21]. The fixed-effect model was used because of homogeneity among studies (I^2^ = 0%). Meta-analysis showed that there is no significant difference in PFS when CTLA-4 inhibitors were added to neoadjuvant PD-1/PD-L1 inhibitor for patients with solid malignancies (HR 1.08, 95%CI 0.47–2.49, *P* = 0.87) (Fig. 4b).

RFS: Two studies reported on RFS, involving 67 participants [21, 26]. The fixed-effect model was used because of homogeneity among studies (I^2^ = 0%). Meta-analysis showed that there is no significant difference in RFS when CTLA-4 inhibitors were added to neoadjuvant PD-1/PD-L1 inhibitor for patients with solid malignancies (HR 1.05, 95%CI 0.31–3.54, *P* = 0.94) (Fig. 4c).

#### Grade 3–4 AEs

**Fig. 5:**
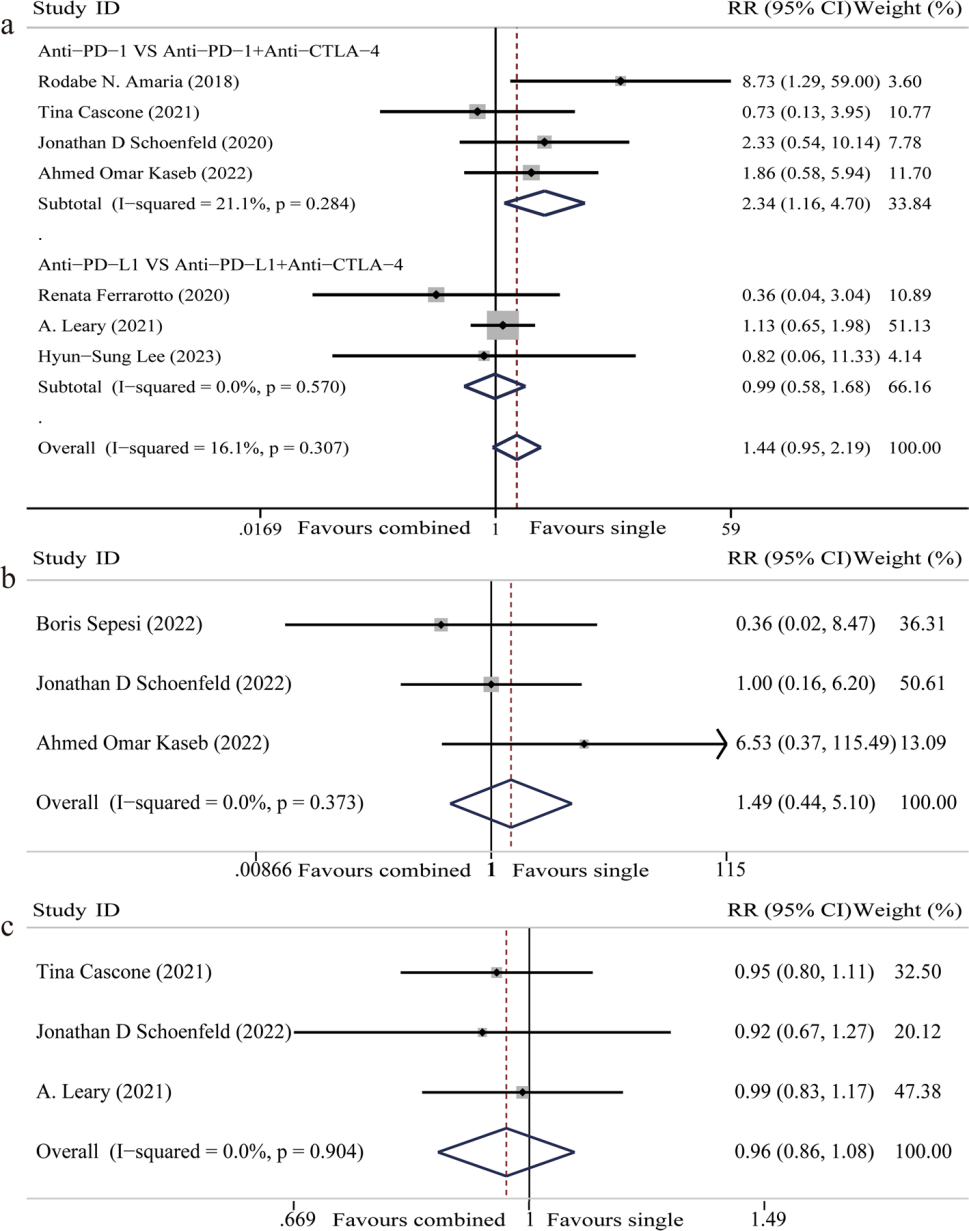
Forest plot of safety. **a** Forest plot of grade 3–4 adverse events. **b** Forest plot of all-cause mortality. **c** Forest plot of completed treatment

Grade 3–4 AEs were reported in seven studies, involving 242 participants [19–21, 26, 28, 29, 31]. The fixed-effect model was used because of homogeneity among studies (I^2^ = 16.1%). Meta-analysis showed that there was no significant difference in grade 3–4 AEs when CTLA-4 inhibitors were added to neoadjuvant PD-1/PD-L1 blockade for patients with solid malignancies (RR 1.44, 95%CI 0.95–2.19, *P* = 0.08) (Fig. 5a). Further subgroup analysis indicated that addition of CTLA-4 inhibitor significantly increased grade 3–4 AEs of patients when PD-1 inhibitor was used (*P* < 0.05), but had no significant impact when PD-L1 inhibitor was used (*P* > 0.05) (Fig. 5a).

#### All-cause mortality

All-cause mortality was reported in three studies, involving 101 participants [20, 25, 27]. The fixed-effect model was used because of homogeneity among studies (I^2^ = 0%). Meta-analysis showed that the addition of CTLA-4 inhibitors had no significant impact on all-cause mortality (RR 1.49, 95%CI 0.44–5.10, *P* = 0.52) (Fig. 5b).

#### Completed treatment

Three studies composed of 143 participants were included [26, 27, 29]. The fixed-effect model was used because of homogeneity among studies (I^2^ = 0%). Meta-analysis showed that there was no significant difference in completed treatment when CTLA-4 inhibitors were added to neoadjuvant PD-1/PD-L1 blockade for patients with solid malignancies (RR 0.96, 95%CI 0.86–1.08, *P* = 0.51) (Fig. 5c).

#### Sensitivity analysis and publication bias

A sensitivity analysis conducted on the included studies, which showed that the results of ORR, MPR, pCR, surgical resection, R0 resection, OS, PFS, RFS, all-cause mortality, and completed treatment remained stable even when each study was removed. However, upon excluding the study by Renata Ferrarotto et al., a change in the result for grade 3–4 AEs was observed, indicating instability in the findings related to grade 3–4 AEs (Fig. S2 and Fig. S3). Furthermore, the egger test and begg test showed no significant publication bias in the analyzed studies(*P* > 0.05) (Table S2).

## Discussion

Antitumor immunity is positively correlated with cellular immunity, primarily mediated by T cells [32, 33]. Activation of cytotoxic T cells relies not only on positive costimulatory signals from the T cell receptor (TCR) binding to tumor antigens, but also on negative coinhibitory signals, such as the PD-1/PD-L1 axis and CTLA-4/B7 axis [34, 35]. Coinhibitory signals become hyperactivated because of overexpressed CTLA-4 in cytotoxic T cells or Treg cells induced by tumors, and overexpressed PD-L1 on tumor cells. Consequently, the activation of T cells is blocked [36, 37]. PD-1/PD-L1 and CTLA-4 inhibitors block the hyperactivated PD-1/PD-L1 axis and CTLA-4/B7 axis respectively, and make effects [11]. Some researchers believe that the overexpressed PD-L1 on cancer cells can suppress the expression of CTLA-4 [38], and the retention of CTLA-4 may weaken the immune activation effect of PD-L1 blockade. Currently, there is controversy regarding whether to block the PD-1/PD-L1 axis and the CTLA-4 simultaneously during neoadjuvant therapy for solid malignancies. Some RCTs have indicated that the additional CTLA-4 blockade is effective and well-tolerated, while others demonstrated that the addition of CTLA-4 inhibitor is inefficient with more adverse events [19–21]. This study included 10 RCTs and shows that adding CTLA-4 inhibitors to PD-1/PD-L1 inhibitors cannot significantly increase ORR, MPR, pCR, surgical resection, prolong the survival time of patients, or improve the safety as neoadjuvant therapy for solid malignancies. Our study supports that the CTLA-4/B7 axis can remain unblocked when treating patients with solid malignancies using neoadjuvant PD-1/PD-L1 inhibitors.

Publications have shown that chemotherapy or radiotherapy can positively affect immune checkpoint inhibitors by inducing inflammatory responses in tumor environments [39–42]. When treating the recurrent/metastatic solid malignancies, the combination of PD-1 or PD-L1 inhibitors and CTLA-4 inhibitors is often used as curative or adjuvant therapy. These agents are often administered prior to or concurrently with traditional chemotherapy or radiotherapy, resulting in favorable clinical outcomes [43, 44]. However, when considering neoadjuvant therapy, only one article was in our study where induced chemotherapy was used before combing PD-1 with CTLA-4 inhibition. Therefore, we speculate that the lack of radiation or chemotherapy induction could be a potential reason for the limited improvement in the efficacy of the additional CTLA-4 inhibition in current studies. Therefore, a combination of traditional radiotherapy or chemotherapy holds promise for enhancing both efficacy and safety. Moreover, individualized combination strategies can be adopted based on differing molecular mechanisms and genomic profiles [45, 46].

In this study, we performed sensitivity analyses and found that the meta-analyses of each efficacy outcome, all-cause mortality, and treatment completion rate remained stable, but the results of grade ≥ 3 AEs were unstable. Additionally, we found that when using PD-1 inhibitors combined with CTLA-4 inhibitors for neoadjuvant therapy for patients with solid maligancies, the grade ≥ 3 AEs significantly increased. This study indicates that the need for further research to investigate whether the combination of neoadjuvant PD-1/PD-L1 inhibitors and CTLA-4 inhibitors leads to an increased rate of AEs.

According to PD-1 or PD-L1 inhibitors, we performed further subgroup analyses. The analyses revealed that the forest plot of ORR and MPR in the PD-1 inhibitor subgroup is more favorable to the experimental group than the PD-L1 inhibitor subgroup, but with no statistical difference. Additionally, the combination of PD-1 and CTLA-4 inhibitors significantly increased the grade 3–4 AEs, whereas PD-1 and CTLA-4 inhibitors did not. When CTLA-4 inhibitors were introduced, this difference between PD-1 and PD-L1 inhibitors may be attributed to the interaction of the PD-1/PD-L1 axis and CTLA-4/B7 axis. Binding of PD-L2 to PD-1 can significantly inhibit CD28 binding to B7 and promote the binding of CTLA-4 to B7 [47]. PD-L1 inhibitors promote the binding of CTLA-4 to B7 and weaken the efficacy of CTLA-4 inhibitors by blocking PD-L1 and retaining PD-L2. On the contrary, PD-L1 and PD-L2 are blocked by PD-1 inhibitors, allowing the efficacy of CTLA-4 inhibitors to remain umimpaired. In addition, CTLA-4 inhibitors can also participate in antibody-dependent cellular cytotoxicity (ADCC) or complement-dependent cytotoxicity (CDC) [48]. Ipilimumab (PD-1 inhibitor) is an antibody of IgG1 that facilitates participation in ADCC or CDC, while tremelimumab (PD-L1 inhibitor) is an antibody of IgG2 which does not engage in these two pathways [49, 50]. This suggests that, compared with the PD-L1 inhibitor, PD-1 inhibitors plus CTLA-4 inhibitors may offer greater efficacy but may slao result in more adverse events.

This study is the first meta-analysis to summarize the efficacy and safety of PD-1 or PD-L1 inhibitors combined with CTLA-4 inhibitors versus mono PD-1 or PD-L1 inhibitors as neoadjuvant therapy for patients with solid malignancies. And it demonstrates that combining PD-1 or PD-L1 inhibitors with CTLA-4 inhibitors is not beneficial. And there are no significant publication biases. However, our study possesses certain limitations. Firstly, further studies are needed in the future because of the small samples and single-center included studies in our study. Secondly, we cannot conduct more detailed subgroup analyses based on different carcinomas due to limited data. Finally, it is important to acknowledge that some HRs of survival were extracted from survival curves, which may introduce potential systematic errors.

## Conclusion

Our study indicates that, as neoadjuvant therapy for solid malignancies, current evidence does not support adding CTLA-4 inhibitors to neoadjuvant PD-1/PD-L1 inhibitors. Moreover, PD-1 inhibitors may be more effective, but potentially increased grade 3–4 adverse events should be concerned.

### Supplementary Information

**Additional file 1.**

## Data Availability

All data generated or analyzed during this study are included in the article or supplementary materials.

## Abbreviations

PD-1: Programmed cell death 1
PD-L1: Programmed cell death ligand 1
CTLA-4: Cytotoxic T Lymphocyte antigen 4
RCTs: Randomized controlled trials
ORR: Overall response rate
MPR: Main pathological response
pCR: Pathological complete response
R0 resection: Radical resection
OS: Overall survival
PFS: Progression-free survival
RFS: Recurrence-free survival
AEs: Adverse events
PICOS: Population, intervention, comparison, outcomes and study design
PRISMA: Preferred Reporting Items for Systematic Reviews and Meta-Analyses
HR: Hazard ratio
CI: Confidence interval
RR: Risk ratios
ROB1: Cochrane Collaboration's tool for assessing risk of bias in randomized trials
TCR: T cell receptor
ADCC: Antibody-dependent cellular cytotoxicity
CDC: Complement-dependent cytotoxicity
