# Supplementary material for: Neoadjuvant PD-1/PD-L1 combined with CTLA-4 inhibitors for solid malignancies: a systematic review and meta-analysis
Source: World J Surg Oncol. 2023 Nov 6;21:349. doi: 10.1186/s12957-023-03212-5 (PMC10626778; doi:10.1186/s12957-023-03212-5)
Supplement: Supplementary file 1 — Additional file 1. [file 12957_2023_3212_MOESM1_ESM.pdf]

World Journal of Surgical Oncology

Neoadjuvant PD-1/PD-L1 combined with CTLA-4 inhibitors for solid malignancies: A systematic review and meta-analysis

Shuang Huang[1], Gang Zheng[2*], Kai Yang[3*]

[1] Department of Stomatology, Shapingba Hospital Affiliated to Chongqing University, No.44, Xiaolongkan New Street, Shapingba District, Chongqing 400030, China

[2] Anorectal Department, Chongqing Traditional Chinese Medicine Hospital, 6 Panxi 7 Road, Jiangbei District, Chongqing 400021, China

[3] Department of Oral and Maxillofacial Surgery, The First Affiliated Hospital of Chongqing Medical University, No.1, Youyi Road, Yuzhong District, Chongqing 400016, China

Correspondence to: cqfyyk@hotmail.com (Kai Yang) and 1045534442@qq.com (Gang Zheng)

* Those authors contributed equally to this work.

Supplementary Material

Supplementary Table

Table S1. Search results of electronic database and manual method.

Database or website	Strategy of searching	Latest retrieval time	Records
Web of Science	((((((((((((TS=("programmed death 1")) OR TS=("pd-1")) OR TS=("programmed death 1 ligand 1")) OR TS=("pd-l1")) OR TS=(immunotherapy)) OR TS=(nivolumab)) OR TS=(pembrolizumab)) OR TS=(toripalimab)) OR TS=(camrelizumab)) OR TS=(durvalumab)) OR TS=(avelumab)) OR TS=(atezolizumab)) OR TS=(tremelimumab)) OR TS=(ipilimumab) AND ((TS=(neoadjuvant)) OR TS=(presurgical)) OR TS=(preoperative) AND (TS=(RCT)) OR TS=(randomized controlled trial)	March 17, 2023	374
PubMed	(PD-1 OR PD-L1 OR CTLA-4 OR immunotherapy OR nivolumab OR pembrolizumab OR toripalimab OR camrelizumab OR durvalumab OR avelumab OR atezolizumab OR tremelimumab OR ipilimumab) AND (neoadjuvant OR presurgical OR preoperative OR perioperative) AND (cancer or carcinoma) AND (RCT OR (randomized controlled trial))	March 17, 2023	322
Embase	('programmed death 1 receptor'/exp OR 'programmed death 1 ligand 1'/exp OR 'pd-1' OR 'pd-l1' OR 'ctla-4' OR 'immunotherapy' OR 'nivolumab' OR 'pembrolizumab' OR 'toripalimab' OR 'camrelizumab' OR 'durvalumab' OR 'avelumab' OR 'atezolizumab' OR 'tremelimumab' OR 'ipilimumab') AND ('neoadjuvant' OR 'presurgical' OR 'preoperative' OR 'perioperative') AND ('cancer' OR 'carcinoma') AND ('rct' OR 'randomized controlled trial')	March 17, 2023	1394
Cochrane Library	(PD-1 OR PD-L1 OR CTLA-4 OR immunotherapy) AND (neoadjuvant OR presurgical OR preoperative OR perioperative) AND (cancer or carcinoma) AND (RCT OR (randomized controlled trial)) in Title Abstract Keyword	March 17, 2023	690
Clinical tral.gov	((PD-1 OR PD-L1 OR CTLA-4 OR immunotherapy) AND (neoadjuvant) in Other Terms	March 17, 2023	NA
ASCO	(PD-1 OR PD-L1 OR CTLA-4 OR immunotherapy) AND (neoadjuvant OR presurgical OR preoperative OR perioperative) AND (cancer or carcinoma) AND (RCT OR (randomized controlled trial))	March 17, 2023	NA
ESMO	(PD-1 OR PD-L1 OR CTLA-4 OR immunotherapy) AND (neoadjuvant OR presurgical OR preoperative OR perioperative) AND (cancer or carcinoma) AND (RCT OR (randomized controlled trial))	March 17, 2023	NA
References of included studies	NA	NA	NA

Abbreviation: NA, not applicable.

Supplementary Figures

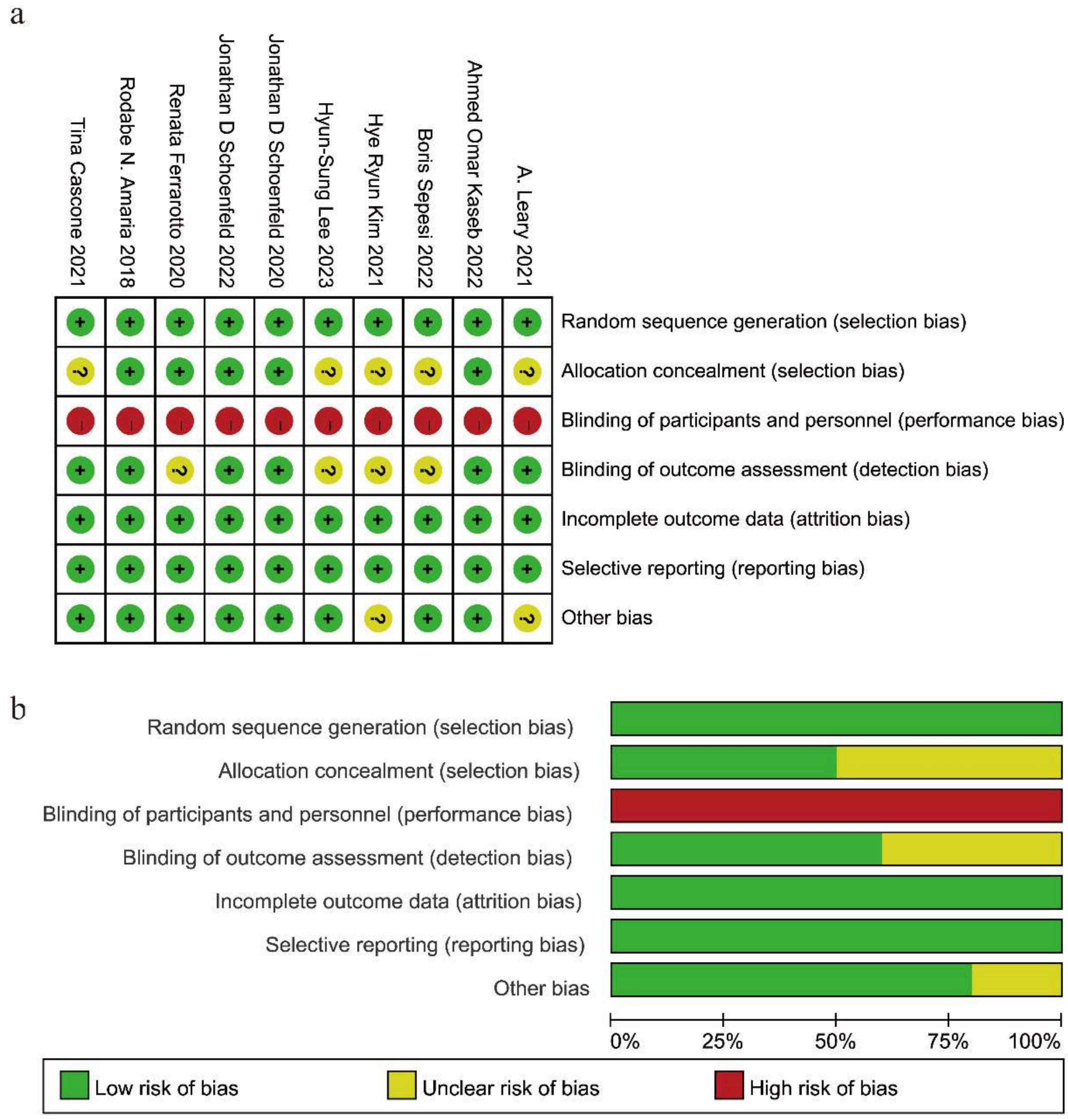

Fig. S1: Risk bias of the included studies. (a) each risk of bias item for included studies. (b) A summary table for each risk of bias item for each study.

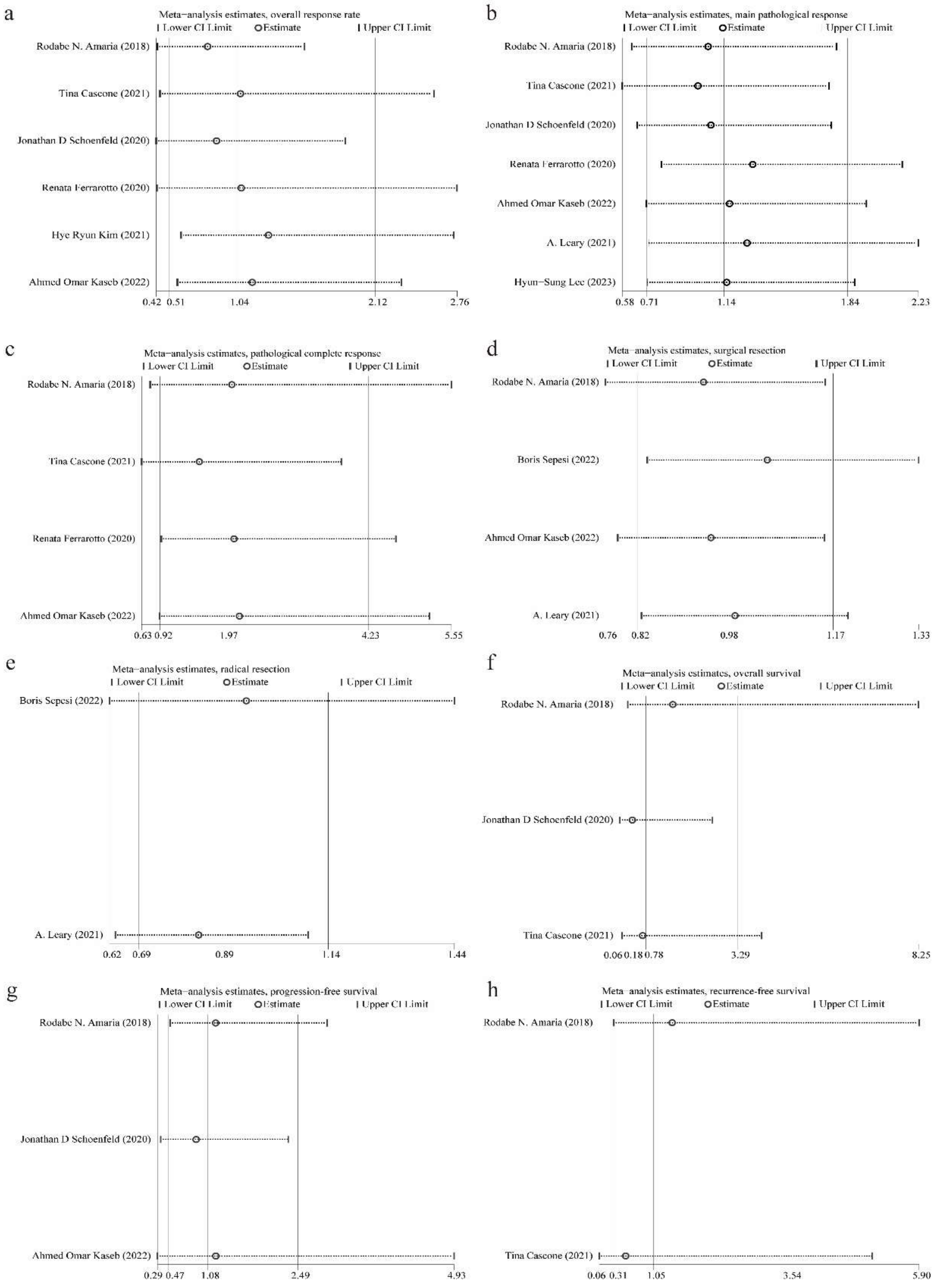

Fig. S2: Sensitivity analyses of efficacy outcomes. (a) Sensitivity analysis of overall response rate (ORR). (b) Sensitivity analysis of main pathological response (MPR). (c) Sensitivity analysis of pathological complete response (pCR). (d) Sensitivity analysis of surgical resection. (e) Sensitivity analysis of residual 0 resection (R0). (f) Sensitivity analysis of overall survival (OS). (g) Sensitivity analysis of progression-free survival (PFS). (h) Sensitivity analysis of recurrence-free survival (RFS).

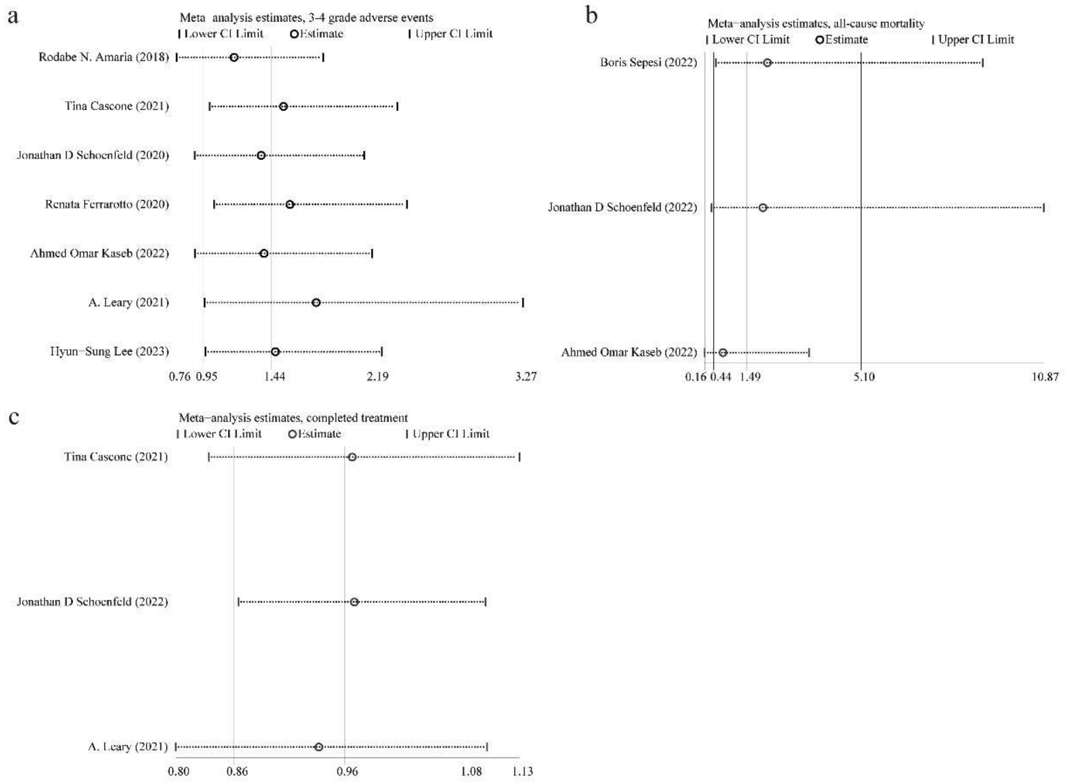

Fig. S3: Sensitivity analyses of safety outcomes. (a) Sensitivity analysis of grade 3-4 adverse events. (b) Sensitivity analysis of all-cause mortality. (c) Sensitivity analysis of completed treatment.
